# mGAP: the macaque genotype and phenotype resource, a framework for accessing and interpreting macaque variant data, and identifying new models of human disease

**DOI:** 10.1186/s12864-019-5559-7

**Published:** 2019-03-06

**Authors:** Benjamin N. Bimber, Melissa Y. Yan, Samuel M. Peterson, Betsy Ferguson

**Affiliations:** 1 Division of Genetics, Oregon National Primate Research Center, Oregon Health and Sciences University, Beaverton, OR 97006 USA; 2 Division of Pathobiology, Oregon National Primate Research Center, Oregon Health and Sciences University, Beaverton, OR 97006 USA; 3 Division of Neuroscience, Oregon National Primate Research Center, Oregon Health and Sciences University, Beaverton, OR 97006 USA; 4 Molecular and Medical Genetics Department, Oregon Health and Sciences University, Portland, OR 97239 USA

**Keywords:** SNP, *Macaca mulatta*, Nonhuman primate, Genome, Rhesus, Indian-origin, Animal model, Database

## Abstract

**Background:**

Non-human primates (NHPs), particularly macaques, serve as critical and highly relevant pre-clinical models of human disease. The similarity in human and macaque natural disease susceptibility, along with parallel genetic risk alleles, underscores the value of macaques in the development of effective treatment strategies. Nonetheless, there are limited genomic resources available to support the exploration and discovery of macaque models of inherited disease. Notably, there are few public databases tailored to searching NHP sequence variants, and no other database making use of centralized variant calling, or providing genotype-level data and predicted pathogenic effects for each variant.

**Results:**

The macaque Genotype And Phenotype (mGAP) resource is the first public website providing searchable, annotated macaque variant data. The mGAP resource includes a catalog of high confidence variants, derived from whole genome sequence (WGS). The current mGAP release at time of publication (1.7) contains 17,087,212 variants based on the sequence analysis of 293 rhesus macaques. A custom pipeline was developed to enable annotation of the macaque variants, leveraging human data sources that include regulatory elements (ENCODE, RegulomeDB), known disease- or phenotype-associated variants (GRASP), predicted impact (SIFT, PolyPhen2), and sequence conservation (Phylop, PhastCons). Currently mGAP includes 2767 variants that are identical to alleles listed in the human ClinVar database, of which 276 variants, spanning 258 genes, are identified as pathogenic. An additional 12,472 variants are predicted as high impact (SnpEff) and 13,129 are predicted as damaging (PolyPhen2). In total, these variants are predicted to be associated with more than 2000 human disease or phenotype entries reported in OMIM (Online Mendelian Inheritance in Man). Importantly, mGAP also provides genotype-level data for all subjects, allowing identification of specific individuals harboring alleles of interest.

**Conclusions:**

The mGAP resource provides variant and genotype data from hundreds of rhesus macaques, processed in a consistent manner across all subjects (https://mgap.ohsu.edu). Together with the extensive variant annotations, mGAP presents unprecedented opportunity to investigate potential genetic associations with currently characterized disease models, and to uncover new macaque models based on parallels with human risk alleles.

**Electronic supplementary material:**

The online version of this article (10.1186/s12864-019-5559-7) contains supplementary material, which is available to authorized users.

## Background

One of the most exciting outcomes from the genomic era and the plunging costs of DNA sequencing is the increased efficiency to identify genetic variants associated with disease risk and progression. This leap forward portends the rapidly approaching era of predictive genetic medicine and personalized therapy. Translating insight into the clinic will require robust genomic models to test and advance gene-based therapeutic approaches. Non-human primates (NHPs), especially macaques, are well positioned to serve as a critical link, providing a highly relevant pre-clinical model. Due to their high level of genomic, physiological, anatomical, neurological and behavioral similarity to humans, macaques are often indispensable to the study of complex human disease.

The close evolutionary history of macaques and humans is evident in their highly similar breadth of natural disease susceptibilities. Moreover, the parallel genetic associations already reported for a broad range of diseases (Table 1) suggests opportunity to leverage the macaque for the discovery of disease-associated biomarkers and for use in the development of new pharmacogenomic or personalized medicine therapies [1–30]. The range of orthologus genetic associations include common diseases, such as cancers, reproductive disorders, retinal and infectious diseases, and rare maladies such as Krabbe disease. Similarly, complex behavioral traits such as heightened anxiety, and variable response to commonly used pharmacological agents such as naltrexone, have been linked to the variants in the same genes as in humans. Other macaque disease models, such as for autism, polycystic ovarian syndrome (PCOS) or type II diabetes, have been reported, but genetic linkages have yet to be uncovered (Table 1). The growing list of macaque traits are likely the ‘tip of the iceberg’ of the natural models of human disease, as other diseases likely go undetected either due to a less obvious phenotype (e.g., hearing loss) or due to early life lethality. The broad spectrum of natural models in rhesus macaque populations presents largely untapped opportunities for translational study, and may negate the need for more costly model development approaches, such as gene-editing.

**Table 1:** Example of diseases and traits reported in rhesus macaques. Diseases are grouped based on whether a genetic association has been reported in macaques for that disease

Genetic Association Reported	
SIV progression [1–3]	
Age-related macular degeneration [4, 5]	
Colorectal cancer [6]	
Krabbe disease [7]	
Ammenorhea [8]	
Naltrexone response [9]	
HPA axis dysregulation [10]	
Alcohol intake levels [11–13]	
Anxiety [14, 15]	
No Genetic Association Reported	
Autism [16]	
Age-associated coginitive decline [17]	
Polycystic ovary syndrome (PCOS) [18]	
Endometriosis [19–21]	
Left ventricular hypertrophy [22]	
Type III Von-Willebrand [23]	
Type II Diabetes [24–26]	
Squamous cell carcinoma [27]	
Coat-like retinopathy [28]	
TB progression [29]	
Osteoporosis [30]	

Despite the clear value of macaques as a human disease models, there are very limited genomic resources available to support the exploration and discovery of potential natural models of genetic disease. Notably, there is a paucity of public databases tailored to searching NHP sequence variant and phenotypic data. The major human variant databases, dbSNP [31] and dbVar [32], phased out support for non-human data in 2017. The NHP Research Consortium Genetic Variant Database website provides a portal for users to deposit NHP SNP data [33]. While valuable, related information about allele frequencies, predicted functional effects, cross-species conservation or links to source animals are not available, limiting the utility for variant interpretation. The Ensembl databases include macaque data, which includes a diverse set of databases and tools, a genome browser, and varying degrees of population-level and phylogenetic data. Here we present the macaque Genotype And Phenotype (mGAP) resource, the first public website providing searchable, extensively annotated macaque genomic data generated from a large, well-characterized rhesus macaque cohort. Once registered, users can browse a curated catalog of variants derived from the whole genome sequence analysis of a large and growing cohort of rhesus macaques. The website and dataset complement existing resources such as Ensembl, primarily by providing data from a defined cohort, much like the 1000Genomes project or similar human cohorts [34]. Importantly, because mGAP provides genotype-level data for all subjects, it is possible to identify specific individuals harboring alleles of interest. We developed a novel annotation pipeline that leverages human annotation data sources to guide interpretation of the macaque variants, an approach that could easily be adapted for other model organisms. The website is designed to allow direct exploration of genomic data, with easy export of all information in standard conventional formats for external processing. mGAP represents an important new resource for NHP genetic and genomic research, which together with the expanding range of macaque ‘omics’ data, supports investigation into links between sequence variants and molecular mechanisms of disease.

## Construction and content

### Rhesus macaque variant catalog

A key feature of mGAP is a catalog of high confidence genomic variants, derived from high depth, whole genome data. The current mGAP release at the time of this publication (1.7) contains whole genome data from 293 Indian-origin rhesus macaques. These animals were selected from a multi-generational outbred cohort of macaques bred at the Oregon National Primate Research Center (average kinship 0.0016). Genomic DNA was isolated, used to generate Illumina libraries as previously described, followed by sequencing on an Illumina XTen sequencer [35]. We obtained an average of 704,193,015 paired Illumina reads per animal, with a range of 549,660,386 - 1,843,458,162 (Additional file 1: Table S1). Primary FASTQ files have been submitted to SRA under BioProject PRJNA382404. All data were aligned to the MMul_8.0.1 genome (GCF_000772875.2), with an average of 700,505,668 paired reads aligned per animal, providing an average high-confidence genome coverage of 20X. These sequence data were processed using a previously described macaque variant calling pipeline ([35] and Additional file 2: Supplemental Methods), adapted from the Broad Institute Best Practice Variant Discovery Pipeline [36]. The pipeline includes extensive quality filtering of the raw variant data as previously described, including the use of Mendelian inheritance for validation.

**Fig. 1:**
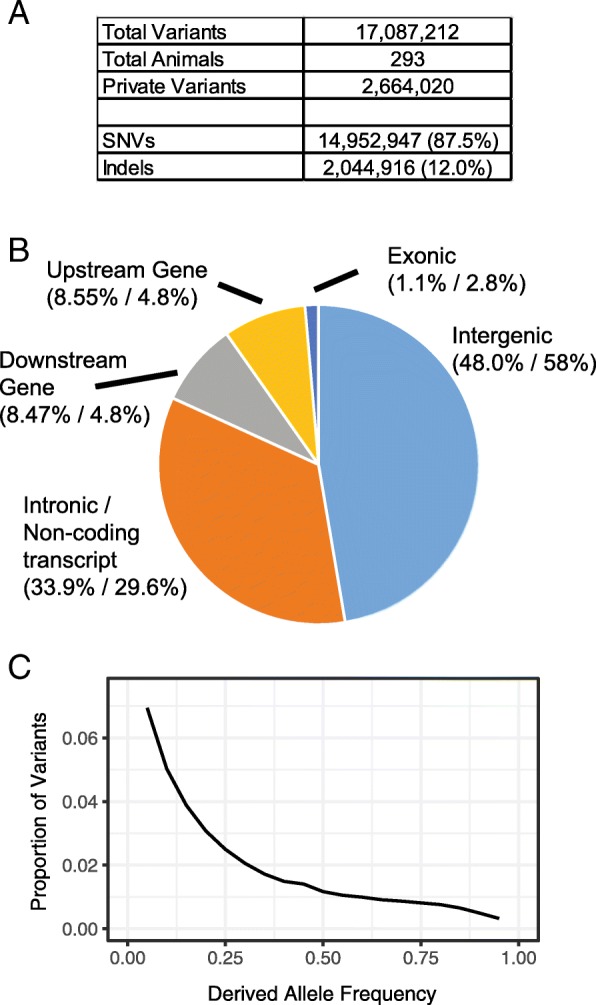
Summary of variant catalog. **a** Table listing the number of total variants and variants by type. **b** Chart showing the distribution of variants relative to gene regions. For each category, the first percentage indicates the fraction of variants of this type. The second percentage indicates the fraction of the genome of this category (when excluding repetitive regions, which are masked from mGAP variant calling). The categories Downstream Gene and Upstream Gene are defined as within 5000 bp of the gene start or end, respectively. **c** The derived allele frequency spectrum of variants in the mGAP database

The mGAP 1.7 release contains 17,087,212 passing variants from this dataset (Fig. 1a). Of these, 2,664,020 (15.5%) are private alleles (detected in only a single individual). Because the cohort includes distantly related individuals, the latter is likely lower than would be observed in an unrelated cohort of similar size. These data include single nucleotide variants (SNVs) and short indels, with the latter comprising 12.0% of variants. As expected, the majority of variants are outside of coding regions (Fig. 1b); however, we identified 250,030 variants within coding regions, including 4888 frameshift mutations and 1871 stop-gained mutations. The mGAP variant database is updated approximately 3–4 times per year, as new data become available.

### Variant functional annotation

**Fig. 2:**
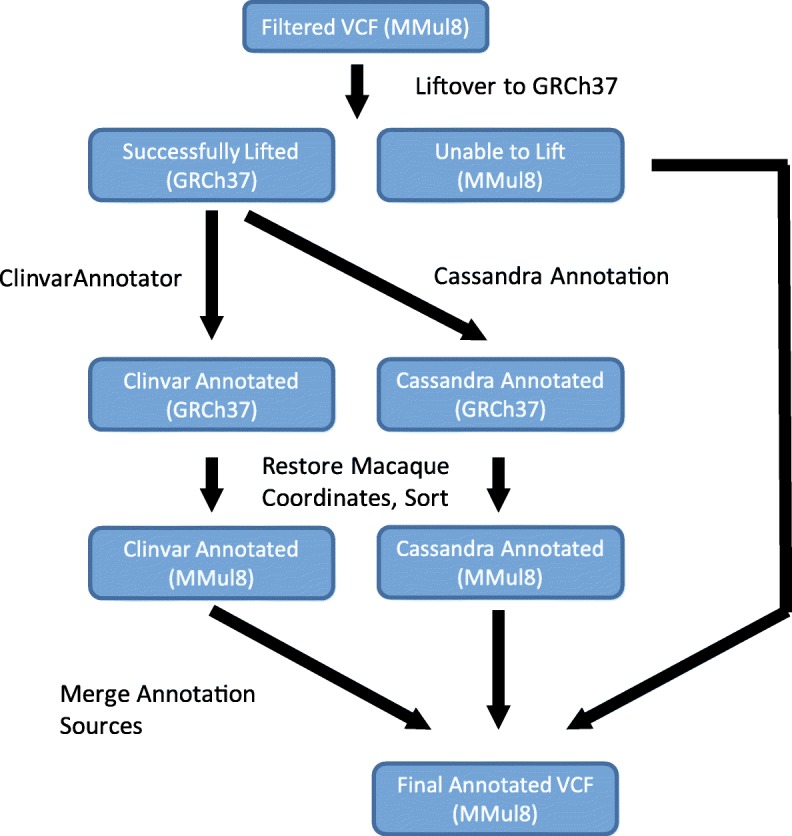
Variant annotation strategy. Macaque variant data are first lifted to the human genome (GRCh37), and annotated against multiple sources. The resulting variants are translated back to the original rhesus macaque coordinates (MMul8.0.1), and merged with the non-lifted variants

Assigning functional significance to genetic variants is often a challenge, and this is particularly problematic for model organisms like macaques that have far less developed annotation resources than human. To overcome this, we developed a multi-part process to annotate our data (Fig. 2). Rhesus macaque variants are first lifted to the human genome (GRCh37.p13, GCA_000001405.14). In the latest release, approximately 65% of macaque variants were successfully lifted to the human genome. Any variant that was unable to lift to the human genome was annotated accordingly. Of the variants that lifted to human, 3,046,012 (17.8%) matched positions reported in the 1000Genomes phase 3 dataset, representing a range of macaque allele frequencies (Additional file 3: Figure S1). The successfully lifted variants were annotated using multiple sources. We utilized Cassandra, a utility that annotates variants overlapping with a variety of human data sources [37], including regulatory elements (ENCODE [38], RegulomeDB [39]), overlap with known disease- or phenotype-associated variants (GRASP [40]), predicted impact (CADD [41, 42], SIFT [43], PolyPhen2 [44]), conservation (Phylop [45], PhastCons [45]), and others [37, 46, 47]. We additionally annotated any variants with identical position and allele as a variant recorded in ClinVar as ‘pathogenic’ or ‘likely pathogenic’ (ClinVar download 9/1/2018). [48–50]. After annotation, variants were translated back to their original macaque genomic coordinates. In addition to human annotations, SnpEff was used to annotate predicted impact on protein coding using the macaque gene annotations [51]. While developed for rhesus macaque, the same liftover annotation process could easily be adapted to other model organisms. The final product of the annotation pipeline is a single annotated VCF file, which is a standard and interchangeable format. All tools created for this process are publicly available [52].

Using this annotation pipeline, there were 2767 variants identical at both position and allele to variants in the human ClinVar database (version GRCh37 20,180,128). Of these, 276 are listed as pathogenic, spanning 258 distinct genes. This list includes variants linked to 160 distinct disorders/phenotypes, including cardiovascular phenotypes (aortic aneurysm, congenital heart disease), immune disorders (polyglandular autoimmune syndrome, Behcet’s syndrome), and many additional rare diseases (Peutz-Jeghers syndrome, Leigh syndrome). Our annotation process also identified many variants predicted to severely impact protein coding, including 12,472 predicted as high impact by SnpEff and 13,129 predicted as damaging by PolyPhen2, encompassing 4091 genes with predicted frameshift mutations. A complete list of variants is available through the mGAP website.

### mGAP website design and data storage

The mGAP website was developed as a module for LabKey Server [53], written using a mixture of Java and JavaScript. The site was designed to heavily leverage existing public resources, such as integration with JBrowse, an open-source genome browser [54]. The source code for the mGAP module and all related modules is freely available through the LabKey Server public subversion repository (https://svn.mgt.labkey.host/stedi/trunk). The primary mode of data storage for each release is the annotated VCF file, which has the advantage of being easily exportable and capable of being processed with most standard variant-processing tools. Limited summary statistics on each release are stored in the database, such as the number of variants and a breakdown of variants by type. The site includes demographics data on all animals, which is also stored and implemented using the LabKey study framework [53].

## Utility and discussion

### The mGAP website overview

**Fig. 3:**
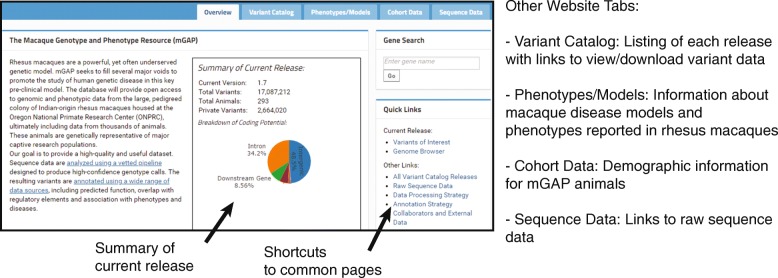
Overview of mGAP website navigation. The front page of the mGAP site contains an overview of the current release, description of the resources, and links to commonly used pages. The website has additional tabs, providing detail on the variant catalog, phenotypes and macaque models, demographics data from the mGAP cohort and access to raw sequence data

The mGAP website provides an interface to download raw data or to explore macaque variants, available at https://mgap.ohsu.edu/. The main page of the website contains a summary of the current release, and tabs for each of the main datatypes: the variant catalog, macaque phenotype and model information, cohort/demographics data and raw sequence data (Fig. 3). The mGAP database currently includes a list of disease models and phenotypes reported in rhesus macaques. The website provides a table with demographics data from the cohort, including sex, species, age, ancestry, and average kinship within the cohort. The mGAP website will be expanded in the future to include clinical data and other phenotypic measures for each subject as well.

### Access to variant data

**Fig. 4:**
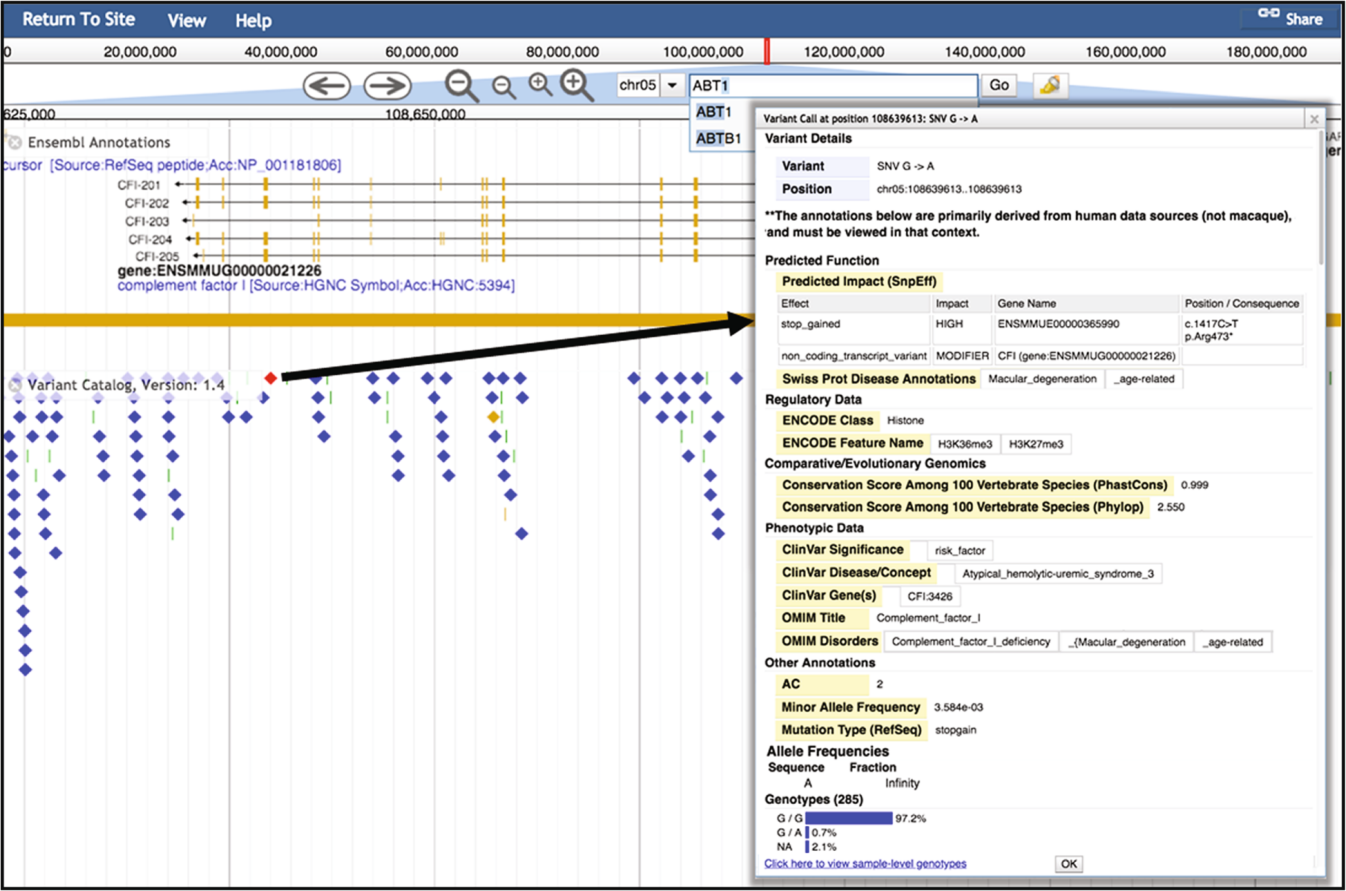
The mGAP genome browser. Variants of interest may be searched by chromosome position or gene name in the search bar. Diamonds indicate SNVs and bars indicate indels, with colors indicating predicted impact on protein coding (red = high, yellow = moderate, blue = low). Clicking a variant provides a detailed pop-up with summary and annotations. The provided information includes predicted function, regulatory data, species conservation, phenotypic data, allele frequencies, and genotypes by animal

The website is designed to allow any user to easily interact with variant data and to support basic queries of the dataset. The genome browser is the primary mode through which users can interactively explore variants. Users can search by genomic position or gene name, and view variants in the context of genes and other macaque genomic features. Variants are color-coded by type and functional prediction to highlight predicted missense and high-impact variants (Fig. 4). Users can click on any variant to view greater detail, including the complete set of functional annotations and cross-species conservation data. The variant details window also includes the minor allele frequency within the currently sequenced population, distribution of homozygous and heterozygous genotypes, and a link to view individual genotypes at this position (as a separate table). In addition, for each release we generate a list of predicted high-impact variants, intended to include those variants either previously implicated in human disease (such as those overlapping ClinVar data), or predicted to have a high impact on protein coding (such as SnpEff high-impact or PolyPhen2 damaging variants). In the 1.7 release, this high-impact list comprises 25,877 genome-wide variants that are predicted to be associated with 2071 human disease or phenotype entries listed in OMIM (Online Mendelian Inheritance in Man).

## Conclusions

The macaque Genotype and Phenotype Resource (mGAP) represents a critical resource for genomic research that fills a major gap in the NHP research field. mGAP provides a unique combination of variant and genotype data from a large cohort of Indian-origin rhesus macaques, processed in a consistent manner across all subjects. While comparable datasets are common for most models, until now they have been lacking for macaques. These data serve basic yet essential functions to support modern genomic analyses, including a catalog of common and rare variants in macaques, coupled with allele frequency and genotype-level data. The mGAP website is designed to allow exploration of data, primarily though the genome browser, or complete export of the entire dataset into common formats for external analysis.

The resources provided by mGAP are designed to advance genomic research in macaques, with the primary goal of identifying and developing new genetic disease models. In this regard, the resource has utility that extends beyond the initial set of sequenced animals. Investigators can explore potential associations between a macaque phenotype of interest and variants in candidate genes by using the mGAP browser to identify compelling candidate variants based on predicted translational effects, allele frequencies and cross species conservation. Prioritized variants can then be evaluated using targeted re-sequencing or genotype-specific assays in macaques lacking genome-wide sequence and tested for phenotype association.

mGAP also presents new opportunities to reveal currently unrecognized macaque disease models, which is facilitated by the highlighted ‘Variants of Interest’ list on the website. This list includes variants that are either identical to those linked to disease in humans or have a predicted highly deleterious impact on protein coding. Supporting this ‘reverse genetic’ approach to model discovery, a comprehensive medical record is established for each of the rhesus macaques housed at the Oregon National Primate Research Center, including regularly updated clinical data, and as available, imaging, histology, and pathology notations. The identification of naturally occurring genetic macaque models provides several advantages over genetically-induced models, such as those produced using CRISPR-like approaches, since there is no danger of experimentally induced off-target effects, the phenotypic evaluation can be carried out in existing animals at considerably less cost than creation of transgenic animals, and the identification of multiple subjects with the desired genotype has the potential to support model expansion.

The rapid expansion of ‘-omic’ level data produced by both NHP and human studies, affords exciting new opportunities for high-resolution data integration and analysis. In this regard, mGAP provides a missing link in the coordinated study of genomic variants in combination with macaque transcriptomic, metabolomic and proteomic findings [55–58]. In parallel, deep learning framework approaches are now emerging that enable the prediction of the functional effects of single base variants on gene expression and disease risk [59]. In addition to purely macaque datasets, studies have shown the value of combined analysis of human and NHP genome-wide data, through the use of neural network approaches that predict variant association with disease, and mGAP data may facilitate similar studies [60].

In addition to model discovery and analysis, mGAP also provides a much-needed resource to improve the design of molecular research tools. For example, information on variant location and allele frequency is critical information to optimize the design of DNA primers for use in PCR amplification or peptide-based ligands for antibody generation. Similarly, SNP array designs for research use or colony management applications will benefit from the available information on allele frequencies and potential pathogenic effects, guiding the selection of appropriate variants to meet the goals of each tool.

Interpretation of genome-wide data can be daunting. For human genomic data, tools continue to be developed to sift through variants and prioritize potentially damaging or relevant variants. The algorithms used in these tools are generally aided by the considerable body of human genome annotation, ranging from catalogs of disease-associated variants to functional annotation developed by experimental studies. Because these resources do not currently exist for macaques, we developed a process to annotate macaque variants using available human data. The annotation processes developed for mGAP can also serve as a template, adapted to enhance the interpretation of genomic variants associated with other model organisms. While lifting human annotation to macaque has value and can identify relevant variants, interpreting annotation across species must be viewed carefully and can have pitfalls, especially for non-coding and regulatory regions. It is therefore important to develop macaque-specific functional annotation, such as data linking genetic variation to changes in gene expression (i.e. eQTLs) or other functional genomic data. The rapid decrease in sequence costs, and rise of massively parallel single-cell analysis approaches, should enable these data to be generated much more rapidly and at a fraction of the cost of the analogous human studies.

While the majority of mGAP data is centered on Indian-origin rhesus macaques from the ONPRC breeding colony, previous studies have found that these macaques are genetically representative of major research captive breeding populations at other US National Primate Research Centers [61]. Further, the utility of mGAP has attracted interest from a broad range of NHP investigators, and thus mGAP now supports data submissions from other rhesus macaque populations. This expansion in data sources will enable immediate cross referencing of variants of interest, facilitating the identification of genotype-specific animals at multiple facilities. In the advancing era of precision medicine, selection and access to genotyped animals will be increasingly critical to enabling state-of-the-art biomedical research.

## Additional files

Additional file 1: **Table S1.** Summary table of per-sample sequence data. (XLSX 27 kb)

Additional file 2: Supplemental Methods. (DOCX 24 kb)

Additional file 3: **Figure S1.** Derived allele frequency spectrum of macaque variants shared with the 1000Genomes Phase 3 dataset. The proportion of variants for each allele frequency is shown for the entire mGAP release 1.7 dataset (blue), and the subset of variants that intersect with sites reported in the 1000Genomes Phase 3 dataset (red). (PDF 69 kb)

## Abbreviations

mGAP: macaque Genotype And Phenotype
NHP: Non-human primate
OMIM: Online Mendelian Inheritance in Man
PCOS: Polycystic ovarian syndrome
WGS: Whole genome sequence

## References

[1] Nomura T, Matano T (2012). Association of MHC-I genotypes with disease progression in HIV/SIV infections. Front Microbiol.

[2] Letvin NL (2011). Immune and Genetic Correlates of Vaccine Protection Against Mucosal Infection by SIV in Monkeys. Sci Transl Med.

[3] Lim SY (2010). Contributions of Mamu-a*01 status and TRIM5 allele expression, but not CCL3L copy number variation, to the control of SIVmac251 replication in Indian-origin rhesus monkeys. PLoS Genet.

[4] Francis PJ (2008). Rhesus monkeys and humans share common susceptibility genes for age-related macular disease. Hum Mol Genet.

[5] Singh KK (2009). Association of HTRA1 and ARMS2 gene variation with drusen formation in rhesus macaques. Exp Eye Res.

[6] Brammer DW (2018). MLH1-rheMac hereditary nonpolyposis colorectal cancer syndrome in rhesus macaques. Proc Natl Acad Sci U S A.

[7] Luzi P (1997). Characterization of the rhesus monkey galactocerebrosidase (GALC) cDNA and gene and identification of the mutation causing globoid cell leukodystrophy (Krabbe disease) in this primate. Genomics.

[8] Lomniczi A (2012). A single-nucleotide polymorphism in the EAP1 gene is associated with amenorrhea/oligomenorrhea in nonhuman primates. Endocrinology.

[9] Vallender EJ (2010). A pharmacogenetic model of naltrexone-induced attenuation of alcohol consumption in rhesus monkeys. Drug Alcohol Depend.

[10] Ferguson B (2012). Genetic load is associated with hypothalamic-pituitary-adrenal axis dysregulation in macaques. Genes Brain Behav.

[11] Lindell SG (2010). Functional NPY variation as a factor in stress resilience and alcohol consumption in rhesus macaques. Arch Gen Psychiatry.

[12] Barr CS (2009). Functional CRH variation increases stress-induced alcohol consumption in primates. Proc Natl Acad Sci U S A.

[13] Cervera-Juanes R (2016). MAOA expression predicts vulnerability for alcohol use. Mol Psychiatry.

[14] Spinelli S (2012). The serotonin transporter gene linked polymorphic region is associated with the behavioral response to repeated stress exposure in infant rhesus macaques. Dev Psychopathol.

[15] Rogers J (2013). CRHR1 genotypes, neural circuits and the diathesis for anxiety and depression. Mol Psychiatry.

[16] Bauman MD, Schumann CM (2018). Advances in nonhuman primate models of autism: Integrating neuroscience and behavior. Exp Neurol.

[17] Paspalas CD (2018). The aged rhesus macaque manifests Braak stage III/IV Alzheimer's-like pathology. Alzheimers Dement.

[18] Abbott DH (2017). Clustering of PCOS-like traits in naturally hyperandrogenic female rhesus monkeys. Hum Reprod.

[19] Assaf BT, Miller AD (2012). Pleural endometriosis in an aged rhesus macaque (Macaca mulatta): a histopathologic and immunohistochemical study. Vet Pathol.

[20] Franasiak JM (2015). Endometrial CXCL13 expression is cycle regulated in humans and aberrantly expressed in humans and rhesus macaques with endometriosis. Reprod Sci.

[21] Zondervan KT (2004). Familial aggregation of endometriosis in a large pedigree of rhesus macaques. Hum Reprod.

[22] Reader JR (2016). Left ventricular hypertrophy in rhesus macaques (Macaca mulatta) at the California National Primate Research Center (1992-2014). Comp Med.

[23] Patterson MM (2002). Type-3 von willebrand’s disease in a rhesus monkey (Macaca mulatta). Comp Med.

[24] Qian C (2015). Diastolic dysfunction in spontaneous type 2 diabetes rhesus monkeys: a study using echocardiography and magnetic resonance imaging. BMC Cardiovasc Disord.

[25] Pare M (2007). Differential hypertrophy and atrophy among all types of cutaneous innervation in the glabrous skin of the monkey hand during aging and naturally occurring type 2 diabetes. J Comp Neurol.

[26] Bremer AA (2011). Fructose-fed rhesus monkeys: a nonhuman primate model of insulin resistance, metabolic syndrome, and type 2 diabetes. Clin Transl Sci.

[27] Jean SM (2011). Spontaneous primary squamous cell carcinoma of the lung in a rhesus macaque (Macaca mulatta). J Am Assoc Lab Anim Sci.

[28] Liu DX (2015). Coats-like retinopathy in a young Indian rhesus macaque (Macaca mulatta). J Med Primatol.

[29] Maiello P, et al. Rhesus Macaques are more susceptible to progressive tuberculosis than Cynomolgus Macaques: a quantitative comparison. Infect Immun. 2018;86(2). 10.1128/IAI.00505-17 PMC5778369 28947646

[30] Simmons HA (2016). Age-associated pathology in rhesus macaques (Macaca mulatta). Vet Pathol.

[31] Sherry ST (2001). dbSNP: the NCBI database of genetic variation. Nucleic Acids Res.

[32] Lappalainen I (2013). DbVar and DGVa: public archives for genomic structural variation. Nucleic Acids Res.

[33] Nonhuman Primate Genetic Variant Database. Available from: https://nprcresearch.org/primate/genetics-genomics/nonhuman-primate-genetic-variant-database.php.

[34] Genomes Project, C (2015). A global reference for human genetic variation. Nature.

[35] Bimber BN (2017). Whole genome sequencing predicts novel human disease models in rhesus macaques. Genomics.

[36] Van der Auwera GA (2013). From FastQ data to high confidence variant calls: the Genome Analysis Toolkit best practices pipeline. Curr Protoc Bioinformatics.

[37] Cassandra: A tool for annotating genomic variant data. Available from: https://www.hgsc.bcm.edu/software/cassandra.

[38] Consortium EP (2004). The ENCODE (ENCyclopedia of DNA elements) Project. Science.

[39] Boyle AP (2012). Annotation of functional variation in personal genomes using RegulomeDB. Genome Res.

[40] Leslie R, O'Donnell CJ, Johnson AD (2014). GRASP: analysis of genotype-phenotype results from 1390 genome-wide association studies and corresponding open access database. Bioinformatics.

[41] Rentzsch P (2019). CADD: predicting the deleteriousness of variants throughout the human genome. Nucleic Acids Res.

[42] Kircher M (2014). A general framework for estimating the relative pathogenicity of human genetic variants. Nat Genet.

[43] Ng PC, Henikoff S (2003). SIFT: predicting amino acid changes that affect protein function. Nucleic Acids Res.

[44] Adzhubei I, Jordan DM, Sunyaev SR (2013). Predicting functional effect of human missense mutations using PolyPhen-2. Curr Protoc Hum Genet.

[45] Ramani R, et al. PhastWeb: a web interface for evolutionary conservation scoring of multiple sequence alignments using phastCons and phyloP. Bioinformatics. 2018. 10.1093/bioinformatics/bty966 PMC6596881 30481262

[46] Consortium EP (2012). An integrated encyclopedia of DNA elements in the human genome. Nature.

[47] Smigielski EM (2000). dbSNP: a database of single nucleotide polymorphisms. Nucleic Acids Res.

[48] Landrum MJ (2018). ClinVar: improving access to variant interpretations and supporting evidence. Nucleic Acids Res.

[49] Landrum MJ (2014). ClinVar: public archive of relationships among sequence variation and human phenotype. Nucleic Acids Res.

[50] Clinvar 2.0 VCF File. Available from: ftp://ftp.ncbi.nlm.nih.gov/pub/clinvar/vcf_GRCh37/archive_2.0/2018/clinvar_20180128.vcf.gz.

[51] Cingolani P (2012). A program for annotating and predicting the effects of single nucleotide polymorphisms, SnpEff: SNPs in the genome of Drosophila melanogaster strain w1118; iso-2; iso-3. Fly (Austin).

[52] DISCVR-Seq. DISCVR-Seq: A set of command line tools for working with sequence data. Available from: https://github.com/BimberLab/DISCVRSeq.

[53] Nelson EK (2011). LabKey server: an open source platform for scientific data integration, analysis and collaboration. BMC Bioinformatics.

[54] Buels R (2016). JBrowse: a dynamic web platform for genome visualization and analysis. Genome Biol.

[55] Lee KJ (2014). Comparative transcriptomics and metabolomics in a rhesus macaque drug administration study. Front Cell Dev Biol.

[56] MacDonald ML (2015). Altered glutamate protein co-expression network topology linked to spine loss in the auditory cortex of schizophrenia. Biol Psychiatry.

[57] Yu X (2016). Quantitative proteomics reveals the novel co-expression signatures in early brain development for prognosis of glioblastoma multiforme. Oncotarget.

[58] Bakken TE (2016). A comprehensive transcriptional map of primate brain development. Nature.

[59] Zhou J (2018). Deep learning sequence-based ab initio prediction of variant effects on expression and disease risk. Nat Genet.

[60] Sundaram L (2018). Predicting the clinical impact of human mutation with deep neural networks. Nat Genet.

[61] Kanthaswamy S (2014). Development and validation of a SNP-based assay for inferring the genetic ancestry of rhesus macaques (Macaca mulatta). Am J Primatol.

