# Supplementary material for: mGAP: the macaque genotype and phenotype resource, a framework for accessing and interpreting macaque variant data, and identifying new models of human disease
Source: BMC Genomics. 2019 Mar 6;20:176. doi: 10.1186/s12864-019-5559-7 (PMC6402181; doi:10.1186/s12864-019-5559-7)
Supplement: Supplementary file 2 — Supplemental Methods. (DOCX 24 kb) [file 12864_2019_5559_MOESM2_ESM.docx]

Supplemental Methods:

*Analysis of Sequence Data and Variant Calling:* Whole genome data were processed using a pipeline following the best practice recommendations from the Broad Institute’s Genome Analysis Toolkit (GATK; [1, 2]), adapted for rhesus macaque. Validation of this pipeline has been previously published [3]. Briefly, paired-end reads were trimmed using Trimmomatic adaptive quality trimming [4], and aligned to the MMul_8.0.1 genome (Assembly ID: GCF_000772875.2), using BWA-MEM [5]. All data were aligned with an average high-confidence genome coverage of 20X (range 12.4-54.8). A list of total reads and average depth per subject is shown in Supplemental Table I. BAM post-processing included local re-alignment around indels using GATK [1], and marking of duplicate reads using Picard tools [6].  GATK’s HaplotypeCaller was used to produce gVCF files, followed by genotype calling using GenotypeGVCFs.  For the latter, a score of 20 was used as the threshold for calling and emitting variants.  The resulting VCF was filtered at the site level using the following criteria: quality by depth (QD < 5.0), strand bias (FS > 15.0), mapping quality (MQ < 50.0), proximity to the read end (ReadPosRankSum < -8.0), the difference in mapping quality between reference and alternate reads (MQRankSum < -12.5), and single nucleotide variant (SNV) clusters of three SNVs within a 10 bp span were also filtered.  In addition, and SNVs located within repetitive regions (identified using RepeatMasker [7] were filtered for removal.  In addition, individual genotypes were filtered if the genotype was supported by fewer than 10X read depth, sites with greater than 100X coverage, or the genotype quality score was less than 20. Many variant calling and genotype errors will occur from regions of the genome that are problematic for short-read sequencing, such as duplicated gene families. In order to identify regions of the genome where our empirical data systematically differs from the reference sequence and are likely to result in genotype errors, we wrote a custom GATK Walker designed to iterate across a series of BAM files and report any positions where three or more distinct alleles are detected in the raw reads of a single subject (tool available on GitHub [8]). These analyses were used to create a blacklist of sites that are also filtered from the final call set. The final dataset includes both SNVs and short indels. The latter include only the default set of indels called by GATK’s HaplotypeCaller/GenotypeGVCFs, and in practice these are limited by the length of Illumina reads, capturing indels approximately 50bp or less. These full analyses also employed Picard tools [6] and FASTQC [9] for quality control of the raw data, and JBrowse [10] to visualize the resulting data. Sequence data were managed and analyzed using DISCVR-Seq [11], a LabKey Server-based system [12].  All pipelines and tools used in this manuscript have been incorporated into DISCVR-Seq [11]. SnpEff was utilized to calculate predicted effects for variants [13]. Ensembl gene annotations were used (release 87). Sequence data were submitted to SRA under BioProject PRJNA340145.

Literature Cited:

1. McKenna, A., et al., *The Genome Analysis Toolkit: a MapReduce framework for analyzing next-generation DNA sequencing data.* Genome Res, 2010. **20**(9): p. 1297-303.

2. Van der Auwera, G.A., et al., *From FastQ data to high confidence variant calls: the Genome Analysis Toolkit best practices pipeline.* Curr Protoc Bioinformatics, 2013. **43**: p. 11 10 1-33.

3. Bimber, B.N., et al., *Whole genome sequencing predicts novel human disease models in rhesus macaques.* Genomics, 2017. **109**(3-4): p. 214-220.

4. Bolger, A.M., M. Lohse, and B. Usadel, *Trimmomatic: a flexible trimmer for Illumina sequence data.* Bioinformatics, 2014. **30**(15): p. 2114-20.

5. Li, H. and R. Durbin, *Fast and accurate long-read alignment with Burrows-Wheeler transform.* Bioinformatics, 2010. **26**(5): p. 589-95.

6. *Picard Tools: A set of command line tools (in Java) for manipulating high-throughput sequencing (HTS) data*. Available from: <http://broadinstitute.github.io/picard/>.

7. Smit, A., Hubley, R & Green, P. *RepeatMasker Open-4.0.* 2013-2015; Available from: <http://www.repeatmasker.org>.

8. DISCVR-Seq. *DISCVR-Seq: A set of command line tools for working with sequence data.*; Available from: <https://github.com/BimberLab/DISCVRSeq>.

9. Andrews, S. *FastQC: a quality control tool for high throughput sequence data*. 2010; Available from: <http://www.bioinformatics.babraham.ac.uk/projects/fastqc>.

10. Skinner, M.E., et al., *JBrowse: a next-generation genome browser.* Genome Res, 2009. **19**(9): p. 1630-8.

11. Bimber, B. *DISCVR-Seq Modules: LabKey Server Extensions for Management and Analysis of Sequencing Data*. 2015; Available from: <https://github.com/BimberLab/discvr-seq/wiki>.

12. Nelson, E.K., et al., *LabKey Server: an open source platform for scientific data integration, analysis and collaboration.* BMC Bioinformatics, 2011. **12**: p. 71.

13. Cingolani, P., et al., *A program for annotating and predicting the effects of single nucleotide polymorphisms, SnpEff: SNPs in the genome of Drosophila melanogaster strain w1118; iso-2; iso-3.* Fly (Austin), 2012. **6**(2): p. 80-92.
