# Supplementary figures and images for: mGAP: the macaque genotype and phenotype resource, a framework for accessing and interpreting macaque variant data, and identifying new models of human disease
Source: BMC Genomics. 2019 Mar 6;20:176. doi: 10.1186/s12864-019-5559-7 (PMC6402181; doi:10.1186/s12864-019-5559-7)

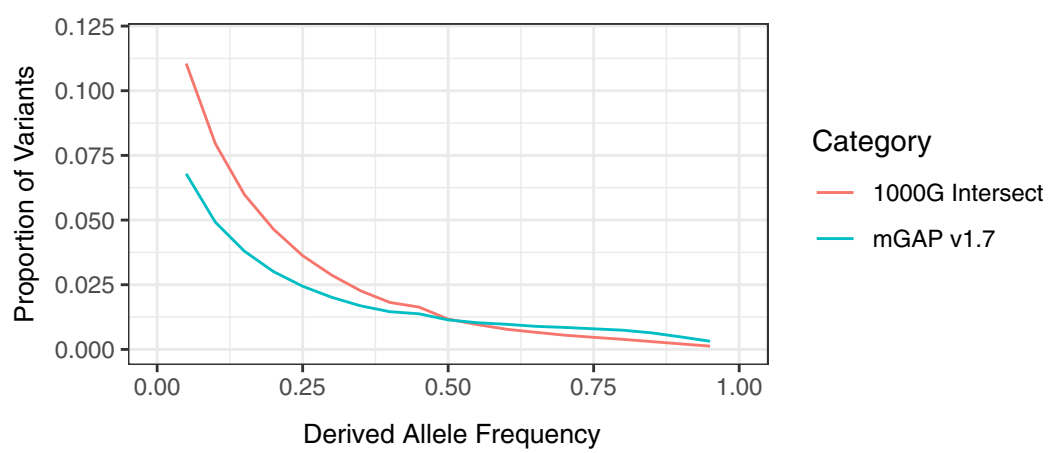

Supplement: Supplementary file 3 — Figure S1. Derived allele frequency spectrum of macaque variants shared with the 1000Genomes Phase 3 dataset. The proportion of variants for each allele frequency is shown for the entire mGAP release 1.7 dataset (blue), and the subset of variants that intersect with sites reported in the 1000Genomes Phase 3 dataset (red). (PDF 69 kb) [file 12864_2019_5559_MOESM3_ESM.pdf]
